# Catamenial chest pain and spontaneous coronary artery dissection: A case report

**DOI:** 10.1016/j.crwh.2020.e00256

**Published:** 2020-09-28

**Authors:** Zainab Al Fatly, Famke L.M. Beckers, Krischan D. Sjauw, Jeanine E. Roeters van Lennep, Michelle M. Schreuder

**Affiliations:** a Department of Internal Medicine, Vascular Medicine, Erasmus Medical Centre, Rotterdam, the Netherlands; b Department of Cardiology, Medical Centre of Leeuwarden, Leeuwarden, the Netherlands

**Keywords:** Spontaneous coronary artery dissection, Menstrual cycle, Estrogen, Cardiovascular disease, Fibromuscular dysplasia, Acute coronary syndrome

## Abstract

Spontaneous coronary artery dissection (SCAD) is a rare cause of myocardial infarction, presenting mostly in healthy, young women. The pathogenesis is still poorly understood. A 45-year-old woman presented with an ST-elevation myocardial infarction, caused by SCAD of the mid left anterior descending coronary artery. In the six years prior to this event, she frequently experienced chest pain coinciding with her menstruation.

## Highlights

• Spontaneous coronary artery dissection in a 45-year-old woman presenting with a ST-elevation myocardial infarction.
• For six years she had experienced chest pain during menstruation.
• The case report highlights the importance of investigating catamenial chest pain.
• Diagnostic tests should be planned and timed according to the menstrual cycle phase in which the chest pain is most severe.

## 1 Introduction

Spontaneous coronary artery dissection (SCAD) is a rare cause of myocardial infarction (MI), presenting mostly in healthy, young women. The exact incidence of SCAD is unknown, but it is estimated to be around 25% of all MIs amongst women aged under 60 years. [1] SCAD is not associated with atherosclerosis or trauma and is not iatrogenic [2]. The dissection may occur in the intima, media or adventitia of the coronary artery wall. It is an underdiagnosed condition and is often missed on a coronary angiogram [3].

We present the case of a woman with SCAD who experienced chest pain related to her menstrual cycle prior to this event.

## 2 Case Presentation

A 43-year-old woman with perimenstrual, episodic chest pain and exertional dyspnea was referred to the cardiology outpatient clinic for cardiac evaluation of suspected coronary artery disease. These chest pains were normally retrosternal, dull and cramping, sometimes with radiation to the throat. They occurred a few days before, during or a few days after menstruation and would last 5–30 min.

She had always been healthy, had had one uncomplicated pregnancy of twins and used no medications. The patient was obese (body mass index: 32,6 kg/m^2^), normal blood pressure (111/73 mmHg), and normal lipid levels (low density lipid cholesterol: 3,0 mmol/L). Furthermore, she did not smoke and had no first-degree family members with cardiovascular disease before the age of 60 years.

She had a normal 12‑lead resting electrocardiogram (ECG) and troponin levels were not elevated. Cardiac ultrasound showed normal cardiac wall motion, normal left- and right-ventricle function and no valve abnormalities. Ergometer stress testing showed no signs of coronary insufficiency. Maximal load was 177 watts (121% predicted), with a heart rate of 173 beats per minute (98% predicted). The conclusion of the cardiologist was that cardiac ischemia, due to obstructive coronary artery disease, was unlikely and the origin of her chest pain either was due to small-vessel disease without clinical consequences, or was stress-related. She was referred back to her general practitioner.

Noteworthy was that the episodes of chest pain always occurred during or a couple of days before or after menstruation. Her menstruation had always been regular, with a duration of vaginal blood loss of 5–10 days. The patient had had severe perimenstrual symptoms, including fluid retention, constipation and migraines, since her menarche.

Six months after the cardiac evaluation, the patient was admitted to the emergency department with severe chest pain, radiating to her jaw, back and neck, with nausea. The pain had started two days after menstruation began. The character of the pain was similar to previous episodes; however, it was more intense and did not subside.

**Fig. 1:**
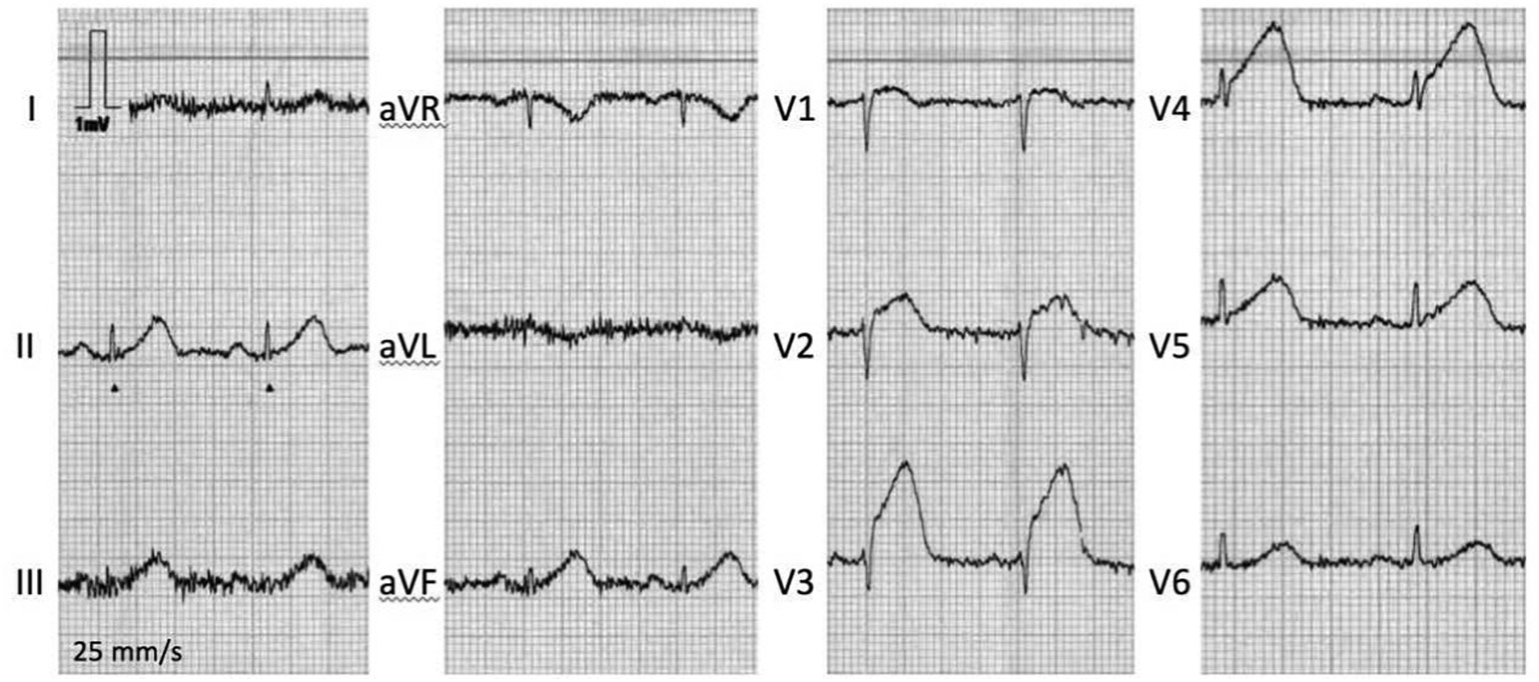
12‑lead ECG in ambulance showing ST-elevations in II, III, aVF, and V1-V5.

**Fig. 2:**
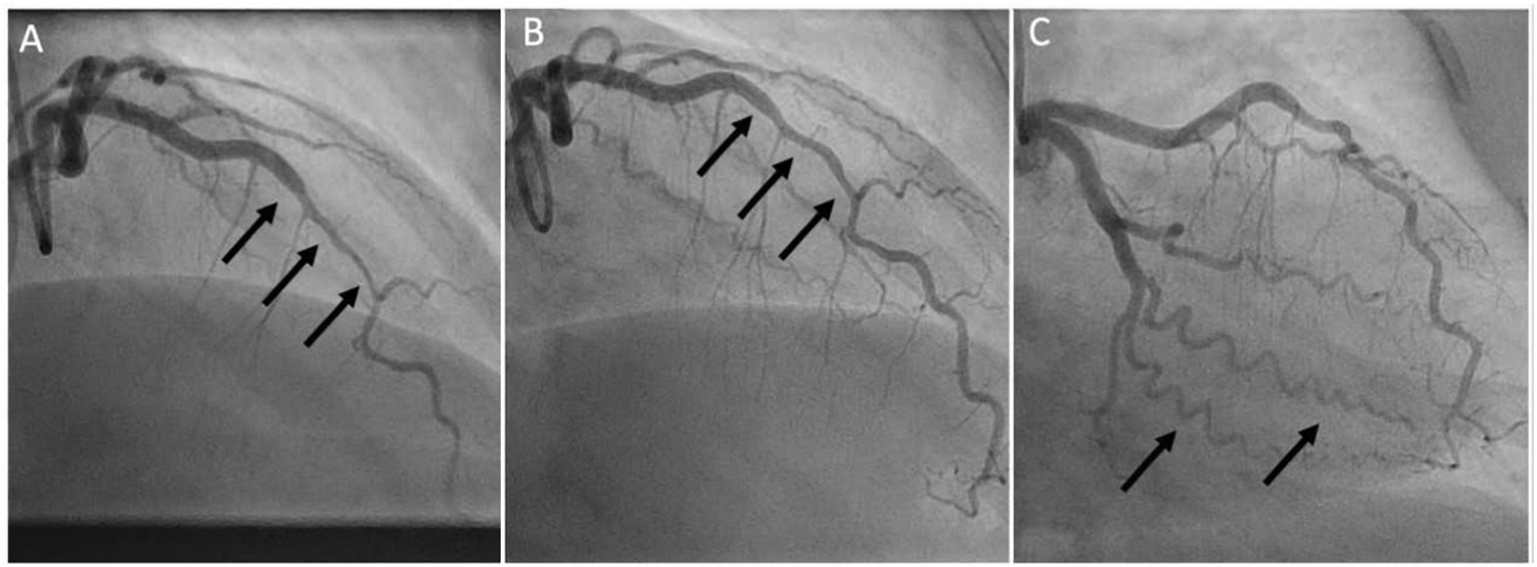
CAG of presented patient. Panel A: a type 2 spontaneous coronary artery dissection (SCAD) within the mid segment of the left anterior descending artery (LAD) at hospital admission with an acute anteroseptal ST-elevation myocardial infarction. Panel B: spontaneous healing of the mid segment of the LAD, 6 weeks after initial presentation. Panel C: typical tortuosity in the coronary arteries often observed patients with SCAD.

A 12‑lead ECG (Fig. 1) showed sinus rhythm at a rate of 77 beats per min and a normal QRS-axis. ST-elevations were observed in leads II, III, aVF, V2-V5, indicative of an acute anterolateral MI. In the ambulance, she was treated according to the ST-elevation myocardial infarction protocol, that is, with aspirin 500 mg, ticagrelor 180 mg and intravenous heparin 5000 IU. Subsequently, ST-elevations resolved completely. At the hospital, coronary angiography (CAG) was performed (Fig. 2). This showed a mid-left anterior descending artery (LAD) lesion, suspected for an intramural hematoma due to SCAD. Furthermore, the coronary arteries seemed to be normal. Coronary vasospasm was ruled out with intracoronary nitroglycerin injection. Percutaneous coronary intervention (PCI) was not performed because of absence of a coronary occlusion, normal coronary flow (Thrombolysis in Myocardial Infarction III), resolution of symptoms and ECG abnormalities during CAG. Moreover, in SCAD a PCI could cause worsening of the dissection. Aspirin, 80 mg once daily, and fraxiparin, 5700 IU twice daily, were started and the patient was discharged. Repeat CAG after 6 weeks showed spontaneous healing of the SCAD lesion.

After discharge the patient was referred to cardiac rehabilitation which she completed in two months. Even though she recovered well, she kept experiencing mild catamenial chest pains, mostly when at rest, at night. To alleviate the pain she was prescribed sublingual nitroglycerin.

A CT scan of the carotid and renal arteries and total aorta was performed to assess fibromuscular dysplasia (FMD) as a cause of SCAD. The scan showed abnormalities of the left renal artery, suggestive of FMD. Also, mild dilatation of the ascending aorta was found (37 mm, 95th percentile of normal). Therefore, the patient was referred to the clinical geneticist to test for mutations related to vascular connective tissue disorders. These were not found. However, absence of genetic mutations [4] does not exclude FMD, as many related mutations are still unknown.

Table 1 provides a timeline of the events in this case.

**Table 1:** Timeline of events.

Time	Event
Since the patient's menarche	Since the patient's menarche, at the age of 11 years, the patient has experienced perimenstrual symptoms. In the previous decade the patient has had multiple episodes of catamenial chest pains, varying in intensity.
Six months prior to the spontaneous coronary artery dissection (SCAD)	Cardiac evaluation. Electrocardiogram showed no abnormalities, the ergometer test showed no signs of cardiac ischemia and cardiac ultrasound showed normal left and right ventricle function and no valve anomalies.
SCAD	Admitted to the emergency room for severe chest pain. Electrocardiogram showed an acute anterolateral ST-elevation myocardial infarction due to a spontaneous dissection of the mid left anterior descending artery and intramural hematoma diagnosed by coronary angiography.
6 weeks after SCAD	Hospital admission for coronary angiography to re-examine the left anterior descending artery dissection, showing the mid-left anterior descending artery had healed well after the SCAD.
Two months after SCAD	CT showed abnormalities of the left renal artery suspect for fibromuscular dysplasia, and mild dilatation of the ascending aorta (37 mm; 95th percentile).

## 3 Discussion

We describe a woman with SCAD, who experienced chest pain that coincided with menstruation. The patient's previous catamenial chest pains were similar to the chest pain she experienced when she had her SCAD, although milder. Recurrent chest pains before the start of menstrual bleeding both before and after experiencing a SCAD have been described earlier, in the setting of variant angina or vasospastic angina [5], and as prodromal to SCAD [6]. There is a lack of evidence for a correlation between catamenial chest pain and SCAD, and so further research is necessary, for example a retrospective study of women who have had a SCAD before or a follow-up study of women with catamenial chest pains.

Low estrogen levels have been associated with cardiovascular disease such as coronary heart disease, migraine and stroke [7]. In small observational studies it has been reported that women have an increased risk of developing chest pain, cardiac ischemia and other vascular-related symptoms around the time of the menstruation, when estrogen levels are low [6].

The patient presented in this case report is suspected to have had FMD, which is related to SCAD. As more than 90% of FMD patients are female, it is likely that sex hormones play a role in the development of FMD [8]. However, the pathways by which female sex hormones, such as estrogen, contribute to the development of FMD have not been identified [9].

There are some limitations to our presentation of the case. The reporting of the episodes of catamenial chest pain was subjective, and there were no recordings of any cardiac abnormalities prior to or during menstruation. Moreover, ischemia might have been missed due to the timing of the cardiac diagnostic procedures, as they were not performed during menstruation, when the patient experienced most complaints.

## 4 Conclusion

This case report adds to the literature because it discusses in detail the course of catamenial chest pain preceding SCAD. By presenting this case, our aim is to increase awareness of the cardiovascular risk of women with catamenial chest pain, as they might have an increased risk for SCAD. These patients might require extra surveillance when reaching the (*peri*)menopause, as this could increase their risk of cardiac ischemia due to decreasing estrogen levels. Moreover, it is important to take catamenial cardiac symptoms seriously and to time diagnostic tests according to the phase of the menstrual cycle when the patient experiences most complaints.
